# HDNetDB: A Molecular Interaction Database for Network-Oriented Investigations into Huntington’s Disease

**DOI:** 10.1038/s41598-017-05224-0

**Published:** 2017-07-12

**Authors:** Ravi Kiran Reddy Kalathur, José Pedro Pinto, Biswanath Sahoo, Gautam Chaurasia, Matthias E. Futschik

**Affiliations:** 1 SysBioLab, Centre for Biomedical Research (CBMR), University of Algarve, Faro, Portugal; 2 TCUBE SOLUTIONS, Bhubaneswar, Odisha India; 3 Institute for Theoretical Biology, Charité, Humboldt-University, Berlin, Germany; 4 Centre of Marine Sciences (CCMAR), University of Algarve, Faro, Algarve Portugal; 5 School of Biomedical and Healthcare Sciences, Plymouth University Peninsula Schools of Medicine and Dentistry, Plymouth, Devon United Kingdom; 6 Department of Biomedicine, University of Basel, Basel, Switzerland

## Abstract

Huntington’s disease (HD) is a progressive and fatal neurodegenerative disorder caused by an expanded CAG repeat in the huntingtin gene. Although HD is monogenic, its molecular manifestation appears highly complex and involves multiple cellular processes. The recent application of high throughput platforms such as microarrays and mass-spectrometry has indicated multiple pathogenic routes. The massive data generated by these techniques together with the complexity of the pathogenesis, however, pose considerable challenges to researchers. Network-based methods can provide valuable tools to consolidate newly generated data with existing knowledge, and to decipher the interwoven molecular mechanisms underlying HD. To facilitate research on HD in a network-oriented manner, we have developed HDNetDB, a database that integrates molecular interactions with many HD-relevant datasets. It allows users to obtain, visualize and prioritize molecular interaction networks using HD-relevant gene expression, phenotypic and other types of data obtained from human samples or model organisms. We illustrated several HDNetDB functionalities through a case study and identified proteins that constitute potential cross-talk between HD and the unfolded protein response (UPR). HDNetDB is publicly accessible at http://hdnetdb.sysbiolab.eu.

## Introduction

Huntington’s disease is an inherited neurodegenerative disorder that results from a trinucleotide (CAG) repeat expansion (>35) in the first exon of the huntingtin (*HTT*, *IT15*) gene^1^. Human HTT codes for a large protein of 3144 amino acids, which is ubiquitously expressed in various tissues and is present in several sub-cellular locations. Studies indicate that both loss of function of normal HTT as well as gain of function of mutant HTT contribute to neuropathological alterations in distinct regions of the brain^2, 3^. In the initial stages of HD, degeneration is detectable mainly in the striatum and cortex, whereas in later stages degeneration is also observed in other brain regions, such as the hypothalamus and hippocampus^4, 5^. Clinically, the disease is characterized by complex and variable symptoms that include movement disorders, psychiatric problems and cognitive decline^2^. Though HD is caused by mutation of a single gene, the disease development might involve a plethora of genes and processes^6^. Indeed, large variations in onset, severity and progression of HD suggest the existence of other influential molecular factors besides the mutation of *HTT* ^7–11^. In order to identify genes that may modify disease onset and progression, genome-wide association and gene expression studies have been performed^12, 13^. Additionally, a large number of genes and proteins have been catalogued based on different types of experimental evidence^6^. Currently, identification of new targets, drugs and therapeutic strategies is at a crucial juncture, which can ultimately contribute to a delayed onset or ameliorate progression of HD.

**Figure 1:**
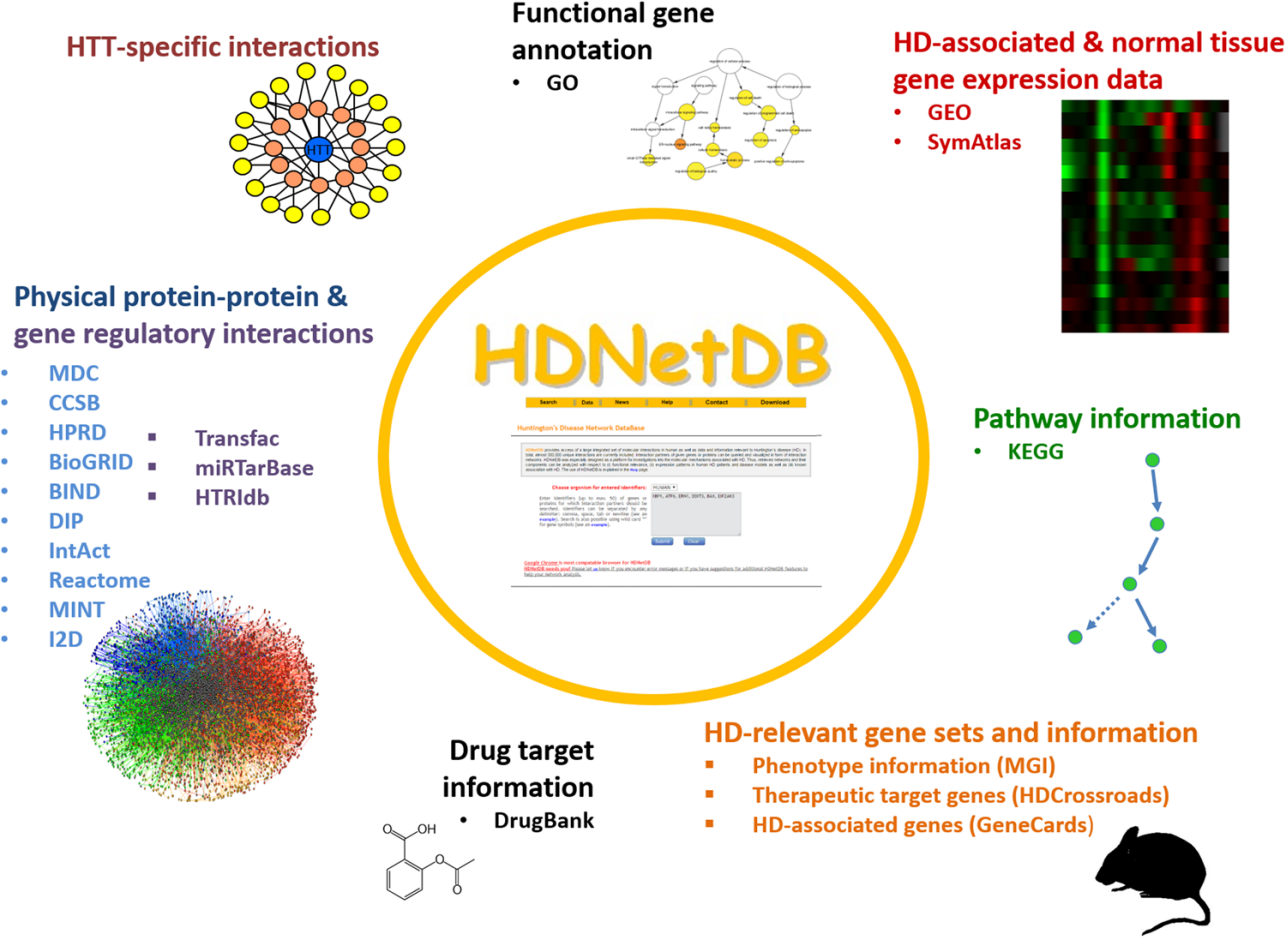
Data and information integrated in HDNetDB. Many types of complementary data and information can be accessed, analyzed and visualized in HDNetDB. While the incorporation of generic data like the human interactome provides a backbone for unbiased network construction, the inclusion of many HD-specific data empower researchers to carry out network-oriented investigations targeting molecular processes in HD.

Despite considerable efforts, deciphering the precise pathological mechanisms underlying HD still requires further research. The large number of genes and the diversity of processes involved in the progression of neurological diseases in general, and HD in specific, emphasizes the need for comprehensive approaches in additional to studies of individual genes^14^. Integrative network models can provide powerful tools for this. The models previously been applied to analyze a wide range of human diseases and have rapidly gained popularity^15–17^. The use of network-based approaches for examining HD is also motivated by the role of HTT. Several studies indicated that HTT interacts with a diverse array of cellular proteins^18–21^. These interacting partners play important roles in various biological processes such as transcriptional regulation, vesicular transport and apoptosis as well as in signaling pathways such as MAPK, mTOR signaling and NOD-like receptor signaling^22, 23^. Thus, it is not surprising that large HTT-focused interaction networks have been derived by independent groups using yeast-two-hybrid (Y2H) screens or affinity purification mass spectrometry (﻿AP﻿-MS)^20, 24–26^. While being formidable approaches, such studies require considerable expertise to assemble and analyze networks, which is a challenging task. In order to assist researchers in their pursuit to understand the disease mechanisms and to identify novel drug targets for HD, we have developed *H*untington’s *D*isease *Net*work *D*ata*B*ase (*HDNetDB*). It constitutes a versatile platform that integrates several levels of data and information ranging from protein-protein interactions, regulatory interactions (microRNA-ta﻿r﻿get gene and transcription factor-target gene), and gene expression to drug-target information about gene, gene ontology and pathway information to phenotype data pertaining to HD (Fig. 1). Besides being a central resource for integrated data and information, HDNetDB also equips users with several querying and visualization options for HD-related networks. HDNetDB is freely accessible at http://hdnetdb.sysbiolab.eu and requires no login. To illustrate the potential of HDNetDB for network-oriented investigations, we describe an exemplary case study focusing on the unfolded protein response (UPR) in the context of HD.

## Results

### Case study: a potential connection between unfolded protein response (UPR) and Huntington’s disease

To illustrate the application of HDNetDB for network-based investigations, we examined the potential connection between HD and UPR, which is a complex intracellular pathway. UPR is activated upon accumulation of unfolded protein in the endoplasmic reticulum (ER). In mammalian cells, the UPR consists of three principal branches defined by signaling components located in the ER membrane: (i) ERN1 (Endoplasmic Reticulum To Nucleus Signaling 1) also referred to as IRE1 (inositol requiring enzyme 1), (ii) EIF2AK3 (Eukaryotic Translation Initiation Factor 2-Alpha Kinase 3) also referred to as PERK (protein kinase R-like ER kinase), and (iii) ATF6 (activating transcription factor 6). The main role of the UPR is to ensure homeostasis by increasing protein folding capacity within the ER, and by reduction of protein synthesis. If homeostasis cannot be re-established, persistent UPR activation can trigger cell death^27^. Although the UPR is well studied, its role in many diseases warrants further elucidation. This is also the case for HD, where different lines of investigation indicated a potential relevance of the UPR for the pathogenesis of HD^28, 29^.

**Figure 2:**
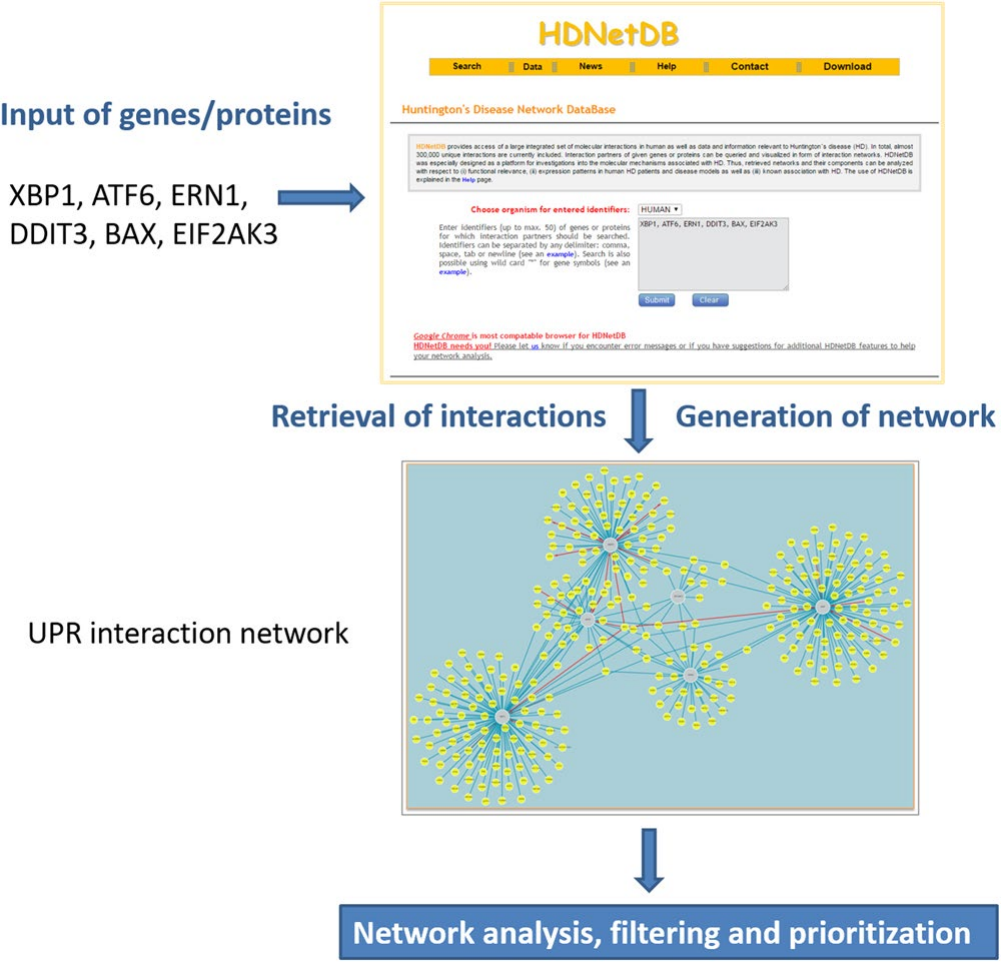
HDNetDB workflow. HDNetDB retrieves the physical and regulatory interactions found for the queried genes or proteins, and generates a network. This is visualized by larger grey and smaller yellow nodes representing the input/query and interacting proteins, while red arrows and blue edges represent regulatory and protein-protein interactions, respectively. Subsequently, the network can be examined and filtered using various complementary datasets and tools integrated in HDNetDB.

To start our network-based investigations, we collected a small set of six key proteins that were reported to be involved in the UPR signaling pathway triggered by ER stress. Besides the three key signaling components mentioned above (ERN1, EIF2AK3, ATF6), we selected transcription factors X-Box Binding Protein 1 (XBP1) and DNA-damage-inducible transcript 3 (DDIT3), also known as CHOP. Both are downstream of ERN1 and EIF2AK3. Additionally, BCL2-associated X protein (BAX) was included, which modulates UPR by a direct interaction with ERN1^30, 31^. It should be emphasized that numerous other proteins have been associated with the UPR, but we took only a small set for better illustration. Nevertheless, we would like to obtain a more comprehensive coverage of proteins associated with UPR and more importantly also of proteins that link the UPR to other processes. Thus, we queried HDNetDB with the six proteins and obtained a set of 354 interacting proteins, which we refer to here as the UPR interactome (Table S1). The workflow and the UPR interactome generated by HDNetDB are presented in Fig. 2. In the network, the six queried proteins serve as “anchor” nodes.

We note that this example shows a common characteristic of network-based investigations: despite starting with a small number of anchor proteins, the retrieved networks are fairly large, especially for well-studied anchor proteins. This makes individual inspection of their components into a highly challenging and time-consuming task. To assist researchers here, HDNetDB offers a series of integrated tools, which enable rapid functional assessment of retrieved networks and prioritization of network components for further investigation.

### KEGG pathway enrichment analysis in UPR interactome

**Figure 3:**
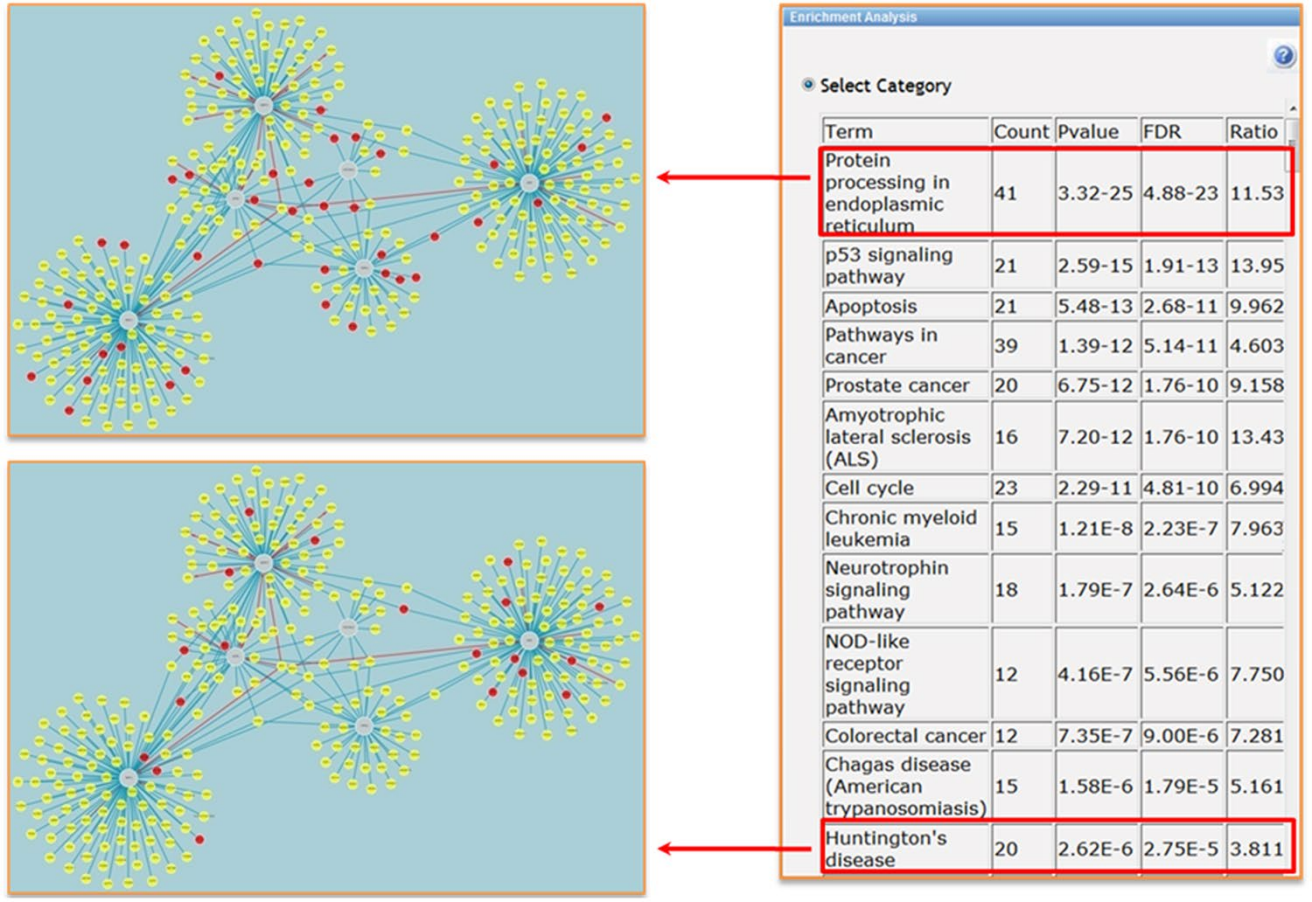
KEGG Pathway enrichment analysis. Results of enrichment analyses are returned as table listing pathways with significant overrepresentation among network proteins (right side). By a mouse click on a table row, components of the selected pathways are highlighted in the network as shown here for “Protein processing in endoplasmic reticulum” and “Huntington’s Disease”.

To gain insights into the functional composition of the retrieved networks, we performed statistical enrichment analysis based on KEGG pathways annotations using the tool implemented on the *Network* page of HDNetDB. This type of analysis can identify those pathways curated in KEGG whose components are significantly overrepresented in the UPR interactome. Thus, we can verify whether we indeed obtained more proteins associated with UPR and we can identify other processes that are linked to the UPR based on the extract interactions. Results of enrichment analysis are returned to the user of HDNetDB as a table listing the detected pathways along with the number of corresponding network proteins and their statistical significance (Fig. 3). For the UPR interactome, the pathway “Protein processing in the ER” achieved expectably the highest significance for overrepresentation (n = 41, FDR = 4.88E^−23^). Notably, apoptosis (n = 23, FDR = 5.48E^−13^) and cell cycle (n = 23, FDR = 2.29E^−10^) were also among the most significant KEGG pathways indicating a tight connection between the UPR and these processes within our network model of the UPR. Strikingly, we also found proteins associated with HD in the KEGG database (n = 20, FDR = 1.88E^−5^) to be strongly overrepresented within the UPR interactome supporting the link between UPR and HD. Besides statistical evaluation, HDNetDB enables the highlighting of proteins associated with the detected pathways and thereby facilitates the individual inspection of pathway components. Examples for this option can be shown in Fig. 3. Alternatively to KEGG annotations, users can carry out functional enrichment analyses based on Gene Ontology (GO) categories for molecular functions, biological processes and cellular compartments.

### Linking the UPR interactome to mammalian phenotypes

**Figure 4:**
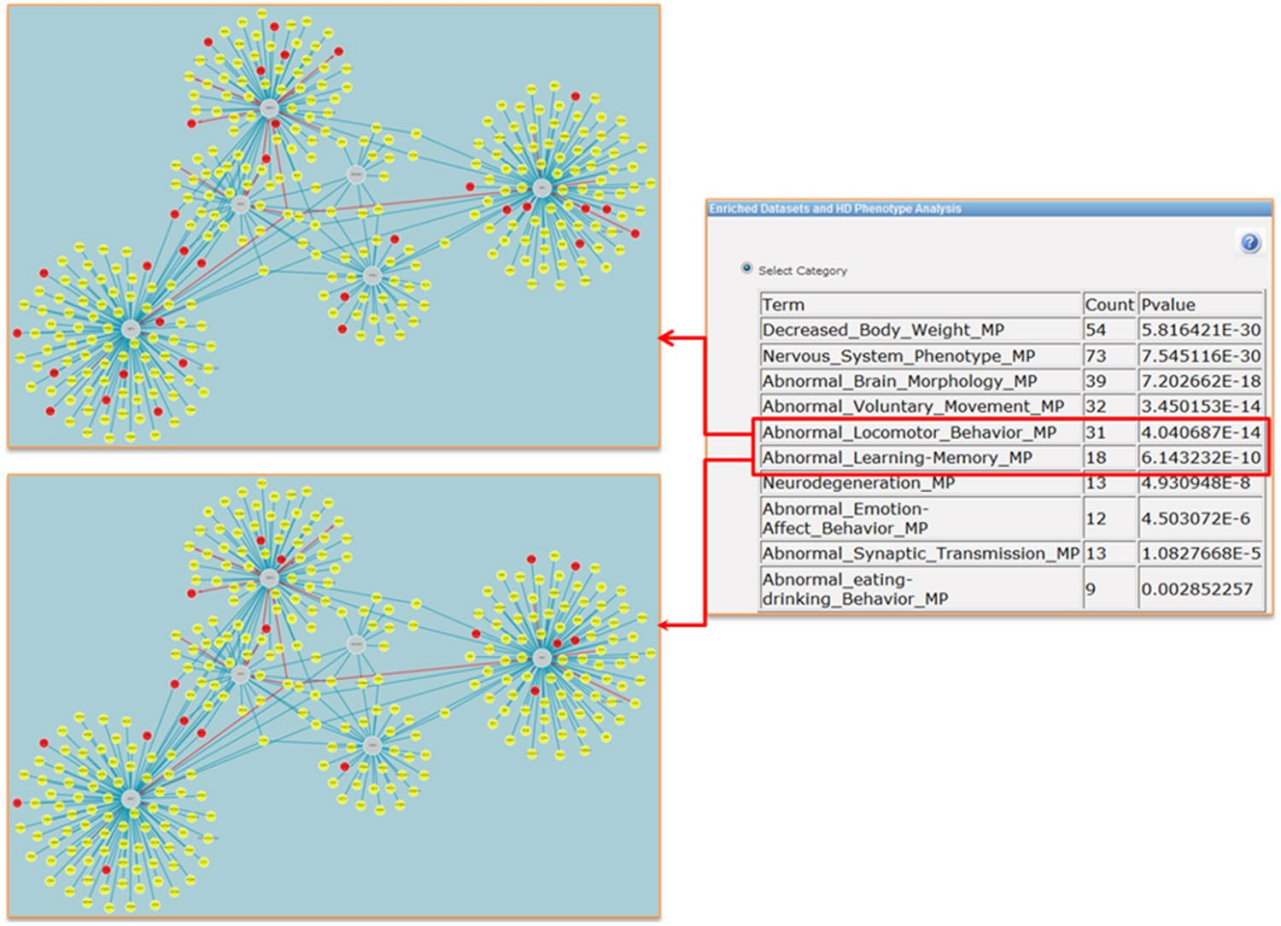
Phenotypic enrichment analysis. HD-relevant mammalian phenotypes are listed, for which a significant enrichment among components of the UPR interactome was detected. Highlighted phenotypes are “Abnormal Locomotor Behavior” and “Abnormal Learning Memory”. Red nodes represent the genes or proteins annotated with these HD-relevant phenotypes in MGI.

Connecting molecular processes to phenotypes is a daunting task in biomedicine. An important help here is provided by extensive cataloging of phenotypes observed for gene knockouts in model organisms. For research into human diseases, the systematic phenotype annotations of murine genotypes provided in the Mouse Genome Informatics (MGI) database are a valuable resource. This is also the case in our study of the UPR interactome. Using the relevant tool implemented in HDNetDB, the network was examined for possible enrichment of proteins associated with HD-relevant phenotypes. Remarkably, we found that most of the selected phenotypes are highly overrepresented among the network components (Fig. 4). For instance, the most significant phenotype (n = 54; p = 5.8E-30) was “Decreased body weight”, which is a common characteristics of HD patients already in the early stage of disease^32^. Intriguingly, the UPR interactome is also strongly enriched in components linked to abnormal locomotor behavior (n = 31; p = 4.04E-14), which is a classical hallmark of HD. To our knowledge, such a connection between UPR and loss of motor control has not been put forward so far. All in all, the results of the phenotypic analysis carried out in HDNetDB suggest that the UPR interactome includes many genes whose knockout in mice lead to HD-relevant phenotypes. These genes can be readily identified interactively in HDNetDB (Fig. 4).

### *In silico* screens with curated gene lists

Besides pathways information and functional or phenotypic annotation, HDNetDB includes curated HD-relevant gene lists, which can be used for examination of networks. Overrepresentation of curated genes among network components can readily be assessed. For the UPR network, HDNetDB identified several gene sets as significantly overrepresented (Supplementary Fig. S1). These include HD Therapeutic Target Genes (HDTTG) – a curated set of genes that were previously identified as potential therapeutic targets in HD^6^. Also, 63 HTT-interacting proteins were identified suggesting not only a functional but also a direct physical connection between UPR and (mutant) HTT. This is in line with previous findings that wild-type HTT is crucial for the integrity of the ER^33^. Since the poly-Q expansion results in a distinct binding behavior of mutant compared to wild-type HTT^23, 34–36^, the results of our analyses suggests that the HD-causing mutation might also have a direct impact on the functioning of the UPR through aberrant protein binding. In addition, we identified a large number of genes (n = 94) that have been genetically associated with neurological diseases, supporting a link between UPR and neuropathology in general. Importantly, users can carry out *in silico* screens based on their own uploaded gene lists, so they are not limited to the curated genes lists provided in HDNetDB.

### Sequential filtering for prioritization of candidate genes

**Figure 5:**
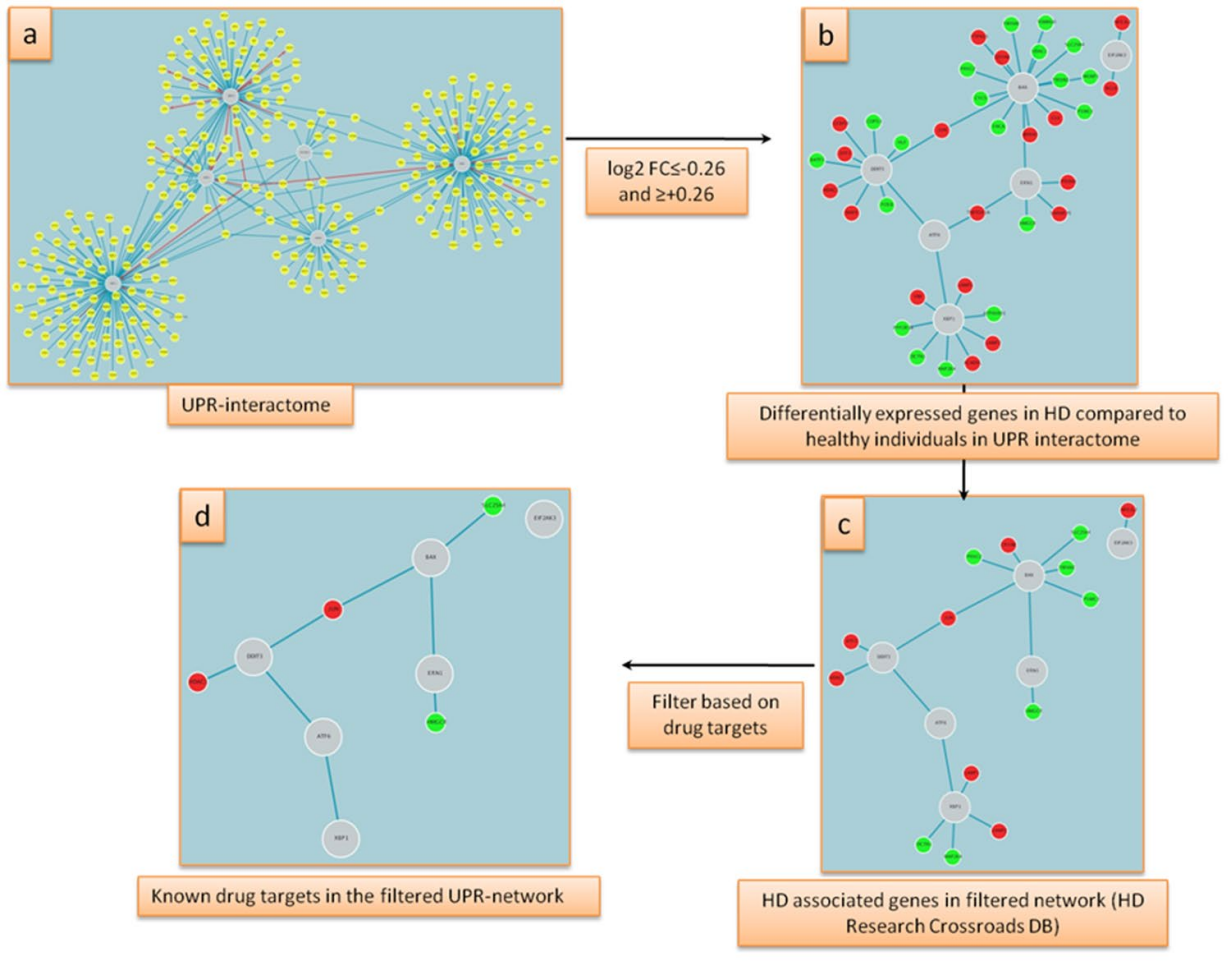
Sequential filtering of UPR interactome. (**a**) Initial UPR network; (**b**) Network after filtering by differential expression in human HD caudate nucleus samples using the criteria log2 fold change ≥ +0.25 and ≤−0.25; (**c**) Network of dysregulated components which have been indicated as potential therapeutic targets for HD; (**d**) Final network obtained after filtering using protein-drug target information (DrugBank). Red and green nodes indicate up and down regulated genes, respectively, and large grey nodes represent query proteins.

Besides the elucidation of the relevance of molecular processes for HD, prioritization of candidate genes for further study and for therapeutic intervention can be carried out efficiently in HDNetDB. Every network produced by application of a filtering procedure can be used as input for another filtering step. In this way, users can define the order and criteria for a sequential filtering procedure in a flexible manner. Moreover, complementary data integrated in HDNetDB can be exploited for network-based gene selection – a strategy which has already been used effectively in molecular pharmacology^37^. For illustration, we carried out step-wise filtering to identify components in the UPR interactome that are (*i*) differentially expressed, (*ii*) associated previously with HD and (*iii*) known drug targets. The underlying motivation for these criteria was to discover proteins related to the UPR whose dysregulation can play a role in the pathogenesis of HD, and can be readily targeted by existing drugs. In the first step, the UPR interactome was filtered based on expression changes between human HD caudate nucleus and normal caudate nucleus, which are available in HDNetDB as one of many comparisons of HD-related gene expression data (Fig. 5a). This resulted in a network of 37 differentially regulated genes, of which 18 are up-regulated and 19 are down-regulated (Fig. 5b). In the next step, we filtered this network based on the criterion that the included components have been either directly or indirectly implicated in HD as described in Kalathur RK *et al*.^6^ (Fig. 5c). This led to the identification of network with 14 genes that are not only differentially expressed in HD but are also implicated in HD and thus may constitute a link between UPR and HD. Finally, we further filtered this network based on known drug-targets present in HDNetDB (Fig. 5d). Only four proteins remained after the sequential filtering that could possibly play key roles in linking HD and UPR, and can be targeted with existing drugs: histone deacetylase1 (HDAC1), jun proto-oncogene (JUN), solute carrier family 25 member 4 (SLC25A4) and 3-hydroxy-3-methylglutaryl-CoA reductase (HMGCR) (Fig. 5d).

**Figure 6:**
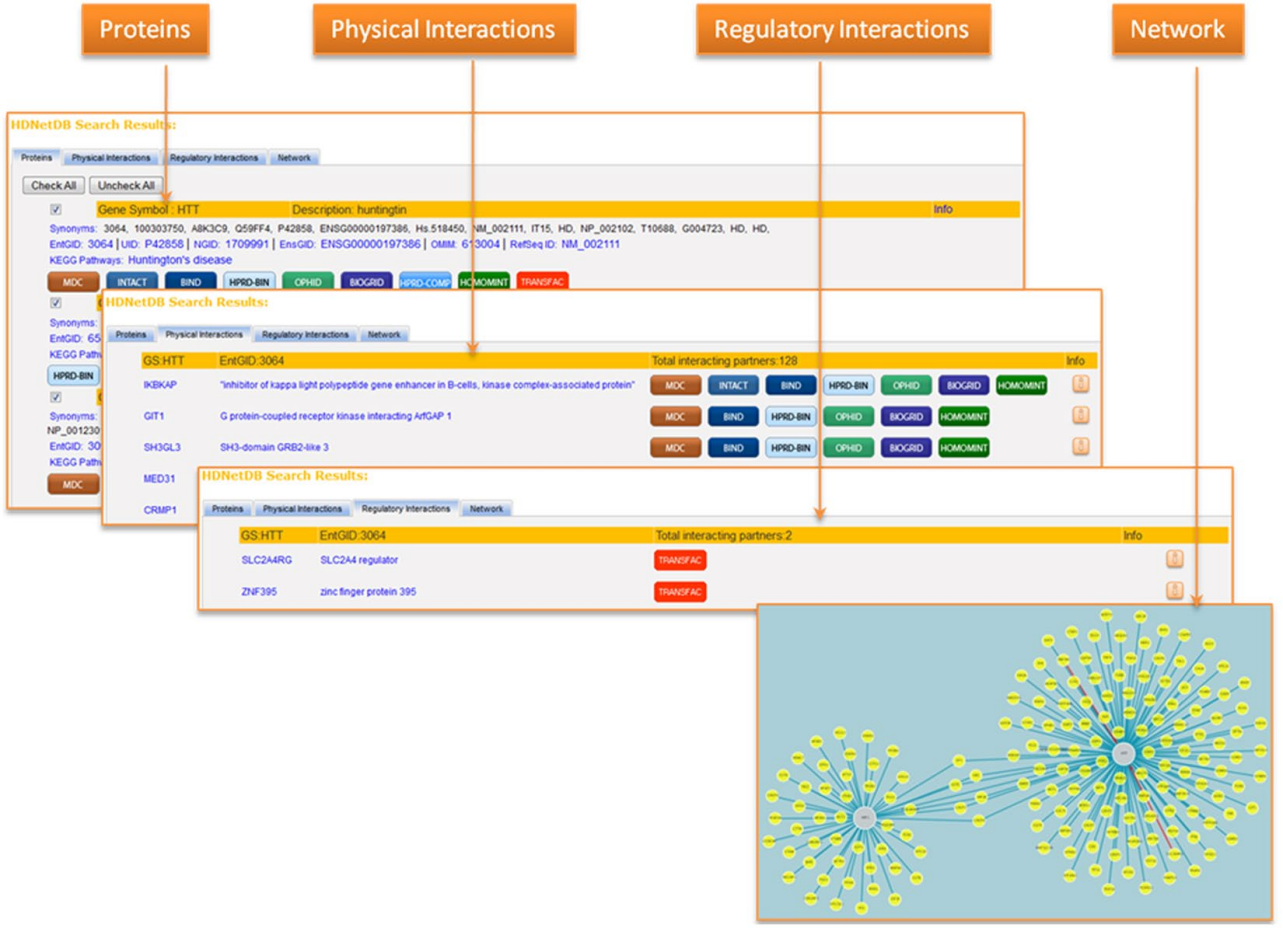
User interface of HDNetDB. After execution of the query, information about proteins and their interactions are shown and visualized on four different pages. The *Network* page provides additionally various tools for interactive analysis.

HDAC1 is a component of the histone deactylase complex and plays an important role in regulation of gene expression. It can act as a molecular switch between neuronal survival and death by interacting with HDRP and HDAC3 respectively^38^. A recent study showed that targeting HDAC1 with HDAC inhibitors resulted in an improvement in HD related phenotypes in different HD model systems^39^. The second gene, *JUN*, is a transcription factor and component of the AP-1 transcription complex that plays a key in role in neural development. Studies have also shown that there is a strong induction of *JUN* both at the gene and protein level in several human neurodegenerative diseases such as Alzheimer’s dementia^40, 41^, Parkinson’s disease^42, 43^ and amyotrophic lateral sclerosis^44^. The third known drug target we identified was SLC25A4 (ANT1), which is a member of a subfamily of solute carrier proteins that help in translocation of ADP from cytoplasm to mitochondrial matrix as well as of ATP from mitochondrial matrix to cytoplasm. In addition, SLC25A4 also regulates the mitochondrial permeability transition pore that initiates apoptosis. It has been speculated that increased expression of *SLC25A4* following a brain injury has biphasic consequence: the initial repair of damaged cells and neurons by increasing ATP export but eventual destruction of damaged cells by apoptosis if the damage is beyond repair^45, 46^. The final gene (*HMGCR*) identified is an ER resident transmembrane glycoprotein and the rate-limiting enzyme for cholesterol synthesis. Perturbations of the cholesterol metabolism have been reported in HD models as well as in HD patients^47^. *HMGCR* levels are regulated in response to sterols by ubiquitin-proteasome system through ER-associated degradation (ERAD) pathway. As mutant HTT has been shown to impair ERAD in cellular models of HD, and thereby to interfere with protein homeostasis in the ER^48^, it is conceivable that an activated UPR might have an impact on the cholesterol synthesis in HD patients. Taken together, the reported findings indicate that the identified genes can provide attractive targets in the context of UPR and HD, although a more comprehensive evaluation is certainly warranted.

## Discussion

HD is a fatal neurodegenerative disease with no known cure. Although it is caused by mutation of a single gene, its molecular manifestation appears to be highly complex and includes numerous processes. To help researchers better cope with the molecular complexity of HD, we have developed HDNetDB. Its development was motivated by our own experiences in network-oriented analyses of HD. Although a large number of tools for analysis of interaction network exists (including our UniHI database^49^), they are generally generic and require laborious data handling to study selected aspects of a specific disease such as HD. HDNetDB can help to overcome these limitations. It is a flexible platform that is customized for HD research. It integrates different types of data ranging from molecular interactions, drug-target information, HD associated genes and their expression in different model organisms and in humans.

HDNetDB was designed to provide easy access to the results of a query. The retrieved data are presented simultaneously on four pages (*Proteins*, *Physical Interactions*, *Regulatory Interactions* and *Network*) enabling the user to switch between different types of information (Fig. 6). The *Proteins* page gives an overview of the genes and proteins matching the query in the database. Proteins, which should not be included as anchor nodes in the generated network, can be excluded. In our case study, for instance, we excluded X-box binding protein 1 pseudogene 1 (XBP1P1). It was found because one of its aliases is the same as the gene symbol of our query protein XBP. Such exclusion on the *Proteins* page will automatically update the network presented on the *Network* page. The *Physical* and *Regulatory Interactions* pages list all interaction partners found at the level of physical protein association or gene regulation for the queried genes and proteins. The sources from which each interaction has been retrieved are shown, and hyperlinks to these sources are provided, if available. In addition, different types of information regarding the individual interaction are given including the methods that were used for identification of the interactions as well as quality scores such as functional co-annotation and co-expression in human tissues. On both *Interactions* pages, options to download the full set of interactions are provided. Finally, the *Network* page displays a graphical visualization of the retrieved network. In addition to simple network visualization, a battery of tools for interactive network analysis is available on the side bar of the *Network* page. First, filtering of interactions can be carried out based on source, type, topology, experimental derivation and number of PubMed references attributed to the interaction. This allows the exclusion of interactions that might be considered less reliable by the user. For instance, setting the required number of PubMed reference to two reduces the network interactions to those that have been reported in at least two publications. Alternatively, interactions from high-throughput experiments or computational prediction can be excluded for network reduction. Second, highlighting and filtering of network components can be performed on the basis of additional information integrated in HDNetDB such as HD-relevant gene expression data, gene sets, and drug target information. Since all filters can be applied sequentially, users of HDNetDB can easily develop and apply their own schemes for prioritization as illustrated in our case study. Third, the functional relevance of displayed networks can be explored through enrichment analysis. HDNetDB calculates the statistical significance of overrepresentation of network proteins among GO categories or KEGG pathways and returns a table with significantly enriched categories or pathways. The genes associated with a detected GO category or pathway can be highlighted in the network by clicking on the specific GO category or pathway in the table. Fourth, an equivalent enrichment analysis can be performed for mammalian phenotypes associated with the network components. These types of enrichment analysis can help to assess the functional relevance of the retrieved networks. Fifth, apart from providing functionalities based on data present in the database, the interface also enables users to map or filter networks based on their own gene list or gene expression data after uploading. In this manner, HDNetDB provides a readily extendable platform for data integration to the user. Lastly, the final network can be exported in a simple text tab-delimited file format, or as an image in PNG or PDF format.

Although many HD-relevant data have been integrated in HDNetDB, researcher might want to carry out analyses beyond the scope of HDNetDB. In this context, HDNetDB can serve as a primary resource for (filtered) interaction networks. Our recent meta-analysis of differential gene expression affecting the UPR in HD provides an example^50^. In that study, we used HDNetDB to define a UPR interactome (in the similar way as presented here), which was then downloaded and further scrutinized using the GSEA software to detect significant trends in expression changes. In addition, we downloaded interactions of apoptosis genes to detect and visualize links between UPR and apoptosis. For this purpose, we used the standalone Cytoscape software due to the large number of interactions, which prohibits a visualization via the web-application. Two possibilities of downloading HDNetDB interaction data in table format exist: (1) the (filtered) network from the *Network* page or (2) the listed interactions on the *Physical* and *Regulatory Interactions* pages. A word of caution here: The first option should only be used for networks with few interactions because of a time-consuming rendering process that underlies the generation of the table on the *Network* page. In contrast, the second option is much more efficient and provides rapid downloads for even large number of interactions. Alternatively, the full set of interaction data integrated in HDNetDB can be downloaded from the webpage.

Since HDNetDB integrates data from different dynamic sources, biannual updates are scheduled. Furthermore, we will continue to incorporate data and information with relevance for HD. These can include data from RNA-seq or genome-wide association studies, or information regarding the permeability of the blood-brain barrier for drugs. To facilitate the generation of high-confidence network, we plan to implement further options for filtering based on co-annotation and co-expression as well as on quality scores provided by some primary resources. An additional feature might be the visual indication of physical complexes in the network.

To conclude, HDNetDB provides an array of tools for querying, analyzing and visualizing molecular interactions. These tools will not only facilitate better understanding of biological processes, functions and pathways that are involved in HD pathogenesis but will also serve to identify novel molecular targets and the development of new therapeutic strategies in curing HD. Finally, we believe that HDNetDB can serve as a general prototype for a new generation of network-oriented resources that are customized towards specific diseases.

## Methods

### Database and architecture

HDNetDB is implemented using platform-independent and open-source software tools. Its architecture comprises two tiers: The database tier consists of a MySQL relational database management system. The application tier complies with an Java 2 Enterprise Edition J2EE architecture and includes Hibernate and Java DataBase Connectivity (JDBC) to connect to the back-end database, Data Access Object (DAO) to interact with the database and accessing data, and JavaServerPages (JSP) to generate web pages. The communication between client and the web application is carried out through Java servlets and JSP, employing an Apache Tomcat server with servlet container.

Molecular networks are visualized using Cytoscape Web library, which enables embedded representation of graphs into a webpage^51^. Performance of Cytoscape Web is a function of graph size, so performance decreases as the number of elements increases. To prevent the visualization tool from becoming unresponsive, certain automatic filtering and layout procedures have been implemented for larger networks (detailed in *Supplementary Materials*).

The HDNetDB database re-utilizes several components that have been developed previously for the Unified Human Interactome (UniHI) database^49^. In contrast to UniHI, which is a generic database for human molecular interactions and their annotation, HDNetDB is tailor-made for HD research and includes many additional datasets and functionalities. In particular, HDNetDB harbors molecular interactions, gene expression data, gene sets and phenotype information associated with HD and neurodegeneration.

### Statistical network analyses

For interpretation of the functional relevance of displayed networks, we have implemented a tool performing enrichment analyses of network proteins for GO annotations^52^ and the public version of KEGG pathways^53^. The statistical enrichment is calculated using the hypergeometric distribution. Enriched terms, along with their significance of enrichment displayed as p-values and false discovery rates (FDR)^54^, are returned to the browser in a tabular format. Computations are performed via Rserve (TCP/IP server), which enables the use of R statistical software and several freely available Bioconductor packages at backend.

### Data integrated in HDNetDB

Various types of data and information have been integrated in HDNetDB. Besides molecular interaction, HDNetDB includes gene expression and drug-target data as well as a diverse range of gene annotations and associations with relevance for neurodegeneration and HD.

### Molecular interaction data

Molecular interactions in HDNetDB comprise both physical protein-protein interactions and regulatory interactions. Currently, HDNetDB integrates about 370000 unique interactions that were derived from large scale studies, literature curation or computational prediction. In particular, physical interactions identified for (mutant) HTT using high-throughput (HTP) Y2H assays or AP-MS were extracted from corresponding publications and merged into one dataset labeled as HTT-HTP^20, 24, 25^. In addition, we imported human protein-protein interactions from MDC^55^, CCSB^56^, HPRD^57^, DIP^58^, BIND^59^, BioGRID^60^, IntAct^61^, REACTOME^62^, COCIT^63^, ORTHO^64^, MINT(HOMOMINT)^65^, I2D (OPHID)^66^ and regulatory interactions from the public version of TRANSFAC^67^, miRTarBase^68^ and HTRIdb^69^ via the UniHI database^49^. Number of proteins and interactions from each source are listed in Table 1.

**Table 1:** Molecular interaction datasets integrated in HDNetDB.

Source	Number of proteins	Number of interactions	Type of interaction	Methods	Reference
HTT-HTP	925	1022	Physical protein interaction	Y2H screens and AP-MS	20, 24–26
MDC-Y2H	1713	3340	Physical protein interaction	Y2H screen	55
CCSB	3741	6821	Physical protein interaction	Y2H screen	56
HPRD	12613	65227	Physical protein interaction	Literature curation	57
BioGRID	14822	124035	Physical protein interaction	Literature curation	58
BIND	11524	19352	Physical protein interaction	Literature curation	59
DIP	3025	2925	Physical protein interaction	Literature curation	85
IntAct	13611	37629	Physical protein interaction	Literature curation	86
Reactome	5315	108867	Physical protein interaction	Pathway curation	87
COCIT	3737	6580	Functional association	Text mining	63
ORTHO	6056	62863	Physical protein interaction	Computational prediction	64
HOMOMINT	6221	21863	Physical protein interaction	Computational prediction + Literature curation	88
OPHID	7874	81677	Physical protein interaction	Computational prediction	89
TRANSFAC	742	1554	Regulatory transcriptional interaction (Transcription factor-based)	Literature curation	67
miRTarBase	2234	3565	Regulatory post-transcriptional interaction (microRNAs-based)	Literature curation	90
HTRIdb	1634	2263	Regulatory transcriptional interactions (Transcription factor-based)	Literature curation	69

The source, number of imported proteins and interactions, type of interaction and methodology used for original identification of the interactions as well as the reference are listed.

### Integrated gene expression data

HDNetDB includes various transcriptome datasets, which can be used to analyze molecular networks. They were obtained from microarray measurements of gene expression undertaken for human HD brain or blood and controls samples as well as for different HD mouse models. An overview of currently included expression datasets is given in Table 2 ^70–76^. Data were obtained from Gene Expression Omnibus and analyzed using the R statistical software and several Bioconductor packages. After preprocessing, log 2 fold changes for different comparisons were calculated from background-corrected and normalized signal intensities and uploaded to HDNetDB. In total, 13 comparisons of expression between HD samples and their corresponding control can be currently accessed. In addition to HD-associated gene expression data, HDNetDB also includes expression profiles for 19 different human tissues obtained from Symatlas^77, 78^.

**Table 2:** Gene expression datasets for human HD samples and mouse models that are integrated in HDNetDB.

#	Species	Tissue/Cell line	Comparison of gene expression	Number of Arrays	Reference
1	Human	Caudate nucleus	Control_vs_HD	404	Hodges *et al*.^70^
			Control_vs_Grade-1 HD		
			Control_vs_Grade-2 HD		
			Control_vs_Grade-3 HD		
2	Mouse	Whole brain	R6-1_18w_vs_wt_18w	18	Hodges *et al*.^71^
			R6-1_22w_vs_wt_22w		
			R6-1_27w_vs_wt_27w		
3	Mouse	Striatum	Ctip2-/-_P0_vs_wt_P0	7	Arlotta *et al*.^72^
4	Mouse	Striatum	R6-2_12 W_vs_wt_12W	26	Kuhn *et al*.^73^
			CHL2-KI_22 M_vs_wt_22M		
5	Mouse	Striatum	YAC-128_12 M_vs_wt_12M	18	Becanovic *et al*.^74^
			YAC-128_24 M_vs_wt_24M		
6	Mouse	Embryonic stem cells	mESC_CAG150_d4_vs_mESC_WT_d4	12	GSE9760
			mESC_CAG150_d6_vs_mESC_WT_d6		
7	Human	Induced pluripotent stem cells (iPSCs)	HD-iPSC_vs_Corrected HD-iPSC	16	An *et al*.^75^
8	Human	Whole blood	HD-Whole-blood_vs_Control-whole-blood	14	Hu *et al*.^76^

The comparisons that can be used for selection and filtering of networks are listed.

### Drug target information

To help rapidly identify known drug targets in molecular networks, relevant information from DrugBank^79^ was imported. Currently, HDNetDB holds information of about 4203 drugs that target about 2139 proteins. In total, 11793 drug target interactions are currently included. If available, the drug’s mode of action can be displayed.

### Gene sets in the context of HD and neurodegeneration

Intersecting networks with informative gene sets can be useful in the prioritization of network components for follow-up studies. At present, five different gene sets associated with HD and neurodegeneration can be mapped onto networks and their statistical enrichment can be calculated in HDNetDB (Table S2): (i) *HDTTG* (*Huntington’s Disease Therapeutic Target Genes*) (n = 950) are comprised of therapeutic target genes described in Kalathur RK *et al*.^6^ and compiled from the HD Research Crossroads database. This gene list was manually curated and each included gene was scored based on existing experimental evidence for a disease-modifying effect. An extended list was recently published and will be included in future versions﻿ of HDNetDB^80^. (ii) *HTT-interacting-proteins* include 1016 genes whose corresponding proteins were detected as directly or indirectly interacting with HTT. This gene set was derived by querying HDNetDB for HTT interactors. (iii) *NDGA* (*Neurological Diseases Gene Association*) consists of 2698 genes that have a genetic association with neurological diseases as indicated in the Genetic Association database^81^. The data were originally extracted from published papers on candidate genes and genome wide association studies. (iv) *HDTM* (*Huntington’s Disease genes through Text Mining*) was derived through text mining implemented in the GeneCards database^82^ using “Huntington’s disease” as a keyword search. The text mining returned 673 genes associated with HD. (v) *NDMOD* (*Neurodegeneration Modifiers*) is derived from independently compiled gene list comprising genetic modifies of neurodegeneration identified in various model systems. The list was created by review of published genetic screens undertaken in *S. cerevisae*, *C. elegans* and *D. melanogaster* models^83^. After mapping to Entrez Gene IDs of the corresponding human orthologs, 217 genes remained.

### Phenotypic information derived from mouse knock-out models

HDNetDB stores genotype-phenotype relations derived from mouse knock-out models for a selected set of phenotypes associated with HD. These were collected from the MGI database^84^ and include nervous system phenotype (MP:0003631; n = 2214), decreased body weight (MP:0001262; n = 1110), abnormal voluntary movement (MP:0003491; n = 904), abnormal locomotor behavior (MP:0001392; n = 850), abnormal brain morphology (MP:0002152; n = 1042), abnormal synaptic transmission (MP:0003635; n = 444), abnormal learning/memory (MP:0001449; n = 413), emotion/affect behavior (MP:0002572 abnormal; n = 346), abnormal eating/drinking behavior (MP:0002069; n = 450) and neurodegeneration (MP:0002229; n = 271). The list of mouse genes obtained for each phenotype are mapped to human orthologs and the corresponding human Entrez Gene IDs are stored in the HDNetDB.

### HDNetDB web-interface: features and functions

As a starting point for a network-based analysis, HDNetDB can be queried for molecular interactions of single or multiple human genes or proteins. As input, identifiers such as gene symbols or Entrez Gene, UniProtKB and Ensembl IDs can be used. Besides identifiers for human genes or proteins, HDNetDB accepts identifiers from model organisms: yeast (*Saccharomyces cerevisiae*), worm (*Caenorhabditis elegans*), fly (*Drosophila melanogaster*) and mouse (*Mus musculus*). These are automatically matched to the corresponding human orthologs to facilitate the practical use of HDNetDB for researchers working with HD model organisms. Accepted identifiers include Entrez Gene IDs for all model organisms, systematic names for yeast, WormBase IDs or gene symbols for worm, FlyBase IDs or gene symbols for fly and MGI identifiers or gene symbols for mouse.

The query generates four pages: *Proteins*, *Physical Interactions*, *Regulatory Interactions* and *Network*. The *Proteins* page lists found genes and proteins in the database. For all matches, the Entrez Gene, UniProtKB, Ensembl gene, RefSeq, and OMIM IDs as well as KEGG pathways (if available) are given along with hyperlinks to the resources. Furthermore, the original interaction datasets, in which the queried genes or proteins were found, are presented. By clicking on the corresponding *Info* button, gene ontology (GO) annotations are additionally displayed. The *Physical* and *Regulatory Interactions* pages show the found interactions and their sources. Detailed information including the method how the interaction was detected, corresponding PubMed references and confidence scores based on GO co-annotations or co-expression can be accessed via a pop-up window through clicking the *Info* button. On the *Network* page, the generated network is displayed. The central nodes corresponding to query genes and proteins are highlighted as grey, large nodes in the default mode, while interacting partners are indicated by yellow, smaller nodes. Blue edges between nodes represent protein-protein interactions, whereas red arrows symbolize regulatory interactions with the given directionality. Using the implemented tools on the side-bar of the *Network* page, the network can be analyzed, sequentially filtered and exported.

## Electronic supplementary material

Dataset 1
