# Supplementary material for: HDNetDB: A Molecular Interaction Database for Network-Oriented Investigations into Huntington’s Disease
Source: Sci Rep. 2017 Jul 12;7:5216. doi: 10.1038/s41598-017-05224-0 (PMC5507972; doi:10.1038/s41598-017-05224-0)
Supplement: Supplementary file 1 — Dataset 1 [file 41598_2017_5224_MOESM1_ESM.doc]

**HDNetDB: A Molecular Interaction Database for Network-Oriented investigations into Huntington's Disease**

Ravi Kiran Reddy Kalathur^1,4^*, José Pedro Pinto^1^, Biswanath Sahoo^2^, Gautam Chaurasia^3^ and Matthias E. Futschik^1,5,6^*

^1^ Centre for Biomedical Research (CBMR), University of Algarve, Faro, Portugal

^2^ TCUBE SOLUTIONS, Bhubaneswar, Odisha, India

^3^ Institute for Theoretical Biology, Charité, Humboldt-University, Berlin, Germany

^4^ Current address: Department of Biomedicine, University of Basel, Switzerland

^5^ Centre of Marine Sciences (CCMAR), University of Algarve, Faro, Portugal

^6^ School of Biomedical and Healthcare Sciences, Plymouth University Peninsula Schools of Medicine and Dentistry, Plymouth, Devon, United Kingdom.

To whom correspondence should be addressed. Tel: +41(0)61 267 35 65; Fax: +41 (0)61 267 35 66; Email: ravikiranreddy.kalathur@unibas.ch

Correspondence may also be addressed to Matthias E. Futschik. Tel: +44(0)1752586458; Email: matthias.futschik@plymouth.ac.uk

**Table S1**. Query proteins and the number of interacting partners found.

| Protein symbol | Protein Name | Functional annotation | Number of interacting proteins |
| --- | --- | --- | --- |
| XBP1 | X-box binding protein 1 | Transcription factor | 102 |
| ATF6 | Activating transcription factor 6 | Transcription factor | 34 |
| ERN1 | Endoplasmic Reticulum To Nucleus Signaling 1 | Ser/Thr kinase with endonuclease activity | 37 |
| DDIT3 (CHOP) | DNA-damage-inducible transcript 3 | Transcription factor | 73 |
| BAX | BCL2-associated X protein | protein binding | 101 |
| EIF2AK3 (PERK) | eukaryotic translation initiation factor 2-alpha kinase 3 | protein serine/threonine kinase activity | 12 |

**Table S2**. List of Huntington's disease specific datasets available in HDNetDB

| Dataset | Number of genes | Source |
| --- | --- | --- |
| HDTTG (Huntington's Disease Therapeutic Target Genes) | 950 | Kalathur et al (6) and HD Research Crossroads |
| HTT-interacting-proteins | 1016 | HDNetDB |
| NDGA (Neurological diseases Gene Association) | 2698 | Genetic Association Database (58) |
| HDTM (Huntington's Disease genes through Text Mining) | 661 | GeneCards database (59) |
| NDMOD (Neurodegeneration Modifiers) | 217 | Chen et al (60) |
| Drug targets | 2139 | DrugBank (57) |
| Mouse phenotype data | 8044 | Mouse Genome Informatics database (61) |

**Supplementary materials**

3) Optimization of network visualization

When the numbers of nodes (interactions) are greater than 400, the network is depicted in radial layout, this takes less computational than the force directed layout which is default layout in HDNetDB. For easy recognition of filtered nodes in the network we retain the specific location of nodes as in the original network after the filtering step, but we also provide a "Recalculate Layout" button to render the visualized network in force directed layout. In the network page HDNetDB will show the network with only those interactions that have more than one PubMed ID associated with when the number of nodes in the networks is greater than 700. Finally user can download full set of interaction for the input from the HDNetDB page.


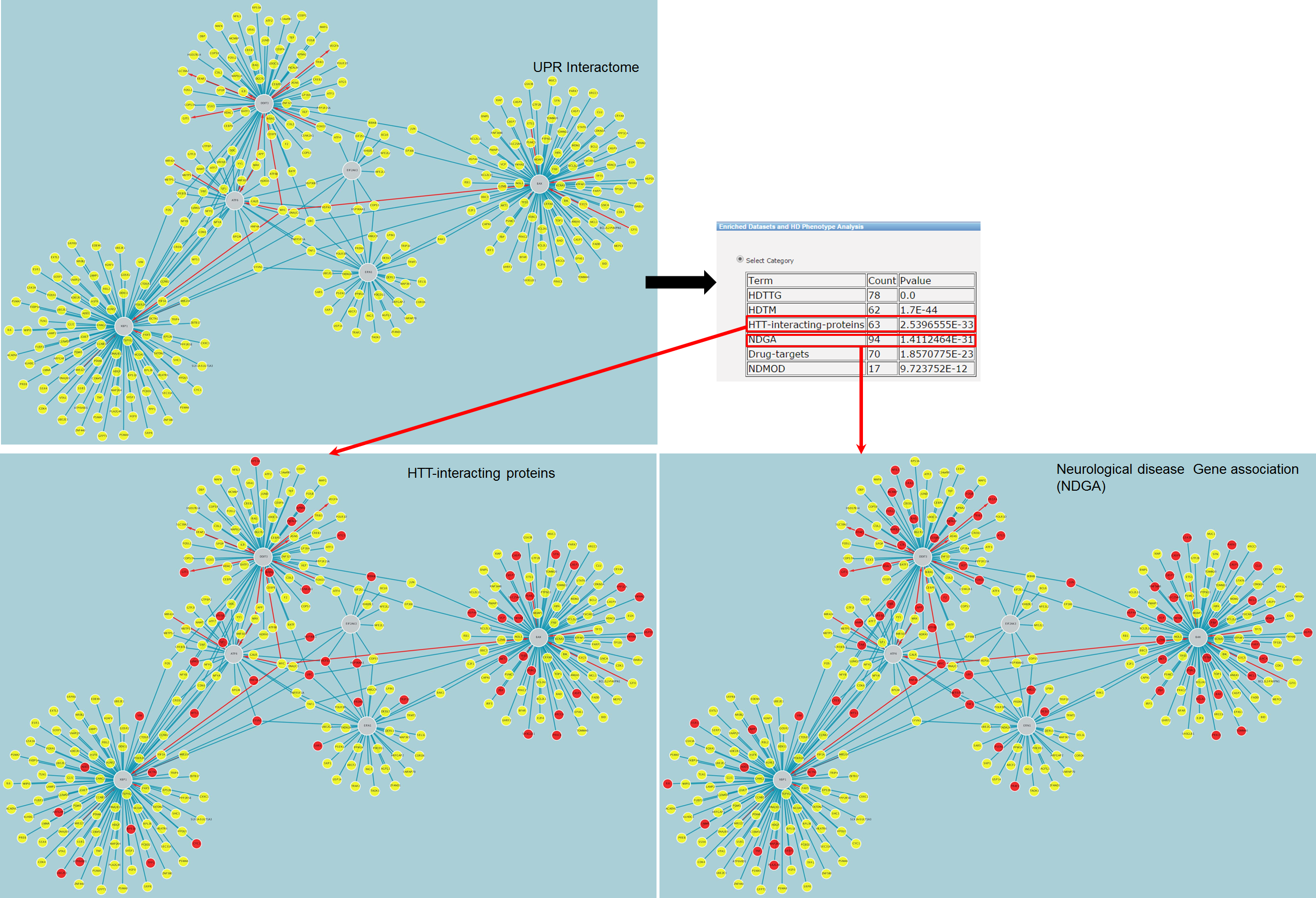


**Fig. S1**. **HD-relevant gene sets enriched in the UPR interactome**. Nodes corresponding to "HTT-interacting proteins (n=63, p-value≤6.3E-29) and “NDGA", (Neurological Disease Gene Association, n=94, p-value≤1.64E-27) are highlighted in red.
